# Assessing the dependence of vehicle rollover on many factors based on new 4D graphs

**DOI:** 10.1371/journal.pone.0284018

**Published:** 2023-04-13

**Authors:** Tuan Anh Nguyen

**Affiliations:** Thuyloi University, Hanoi, Vietnam; Beijing Institute of Technology, CHINA

## Abstract

In this paper, the author proposes a novel solution to evaluate the dependence between the maximum roll angle and the minimum vertical force at the wheel on the vehicle’s height and speed. 4D graphs that fully and clearly describe the dependence between parameters are used to replace conventional 2D and 3D graphs. A complex dynamic model is established to describe the vehicle’s oscillations when steering. Calculations and simulations are performed using Simulink^®^ software with many specific cases. In all cases, the input values such as steering angle, speed, and distance from RA (roll axis) to CG (the center of gravity), all change flexibly. According to the paper’s findings, the roll angle will rise once speed or height increases. In addition, the maximum roll angle increases significantly once both the velocity and the height increase. This causes the vertical force at the wheel to drop suddenly, and rollover may occur if this value reduces to zero. 4D graphs provide a more specific and intuitive assessment of vehicle rollovers.

## 1. Introduction

**Fig 1:**
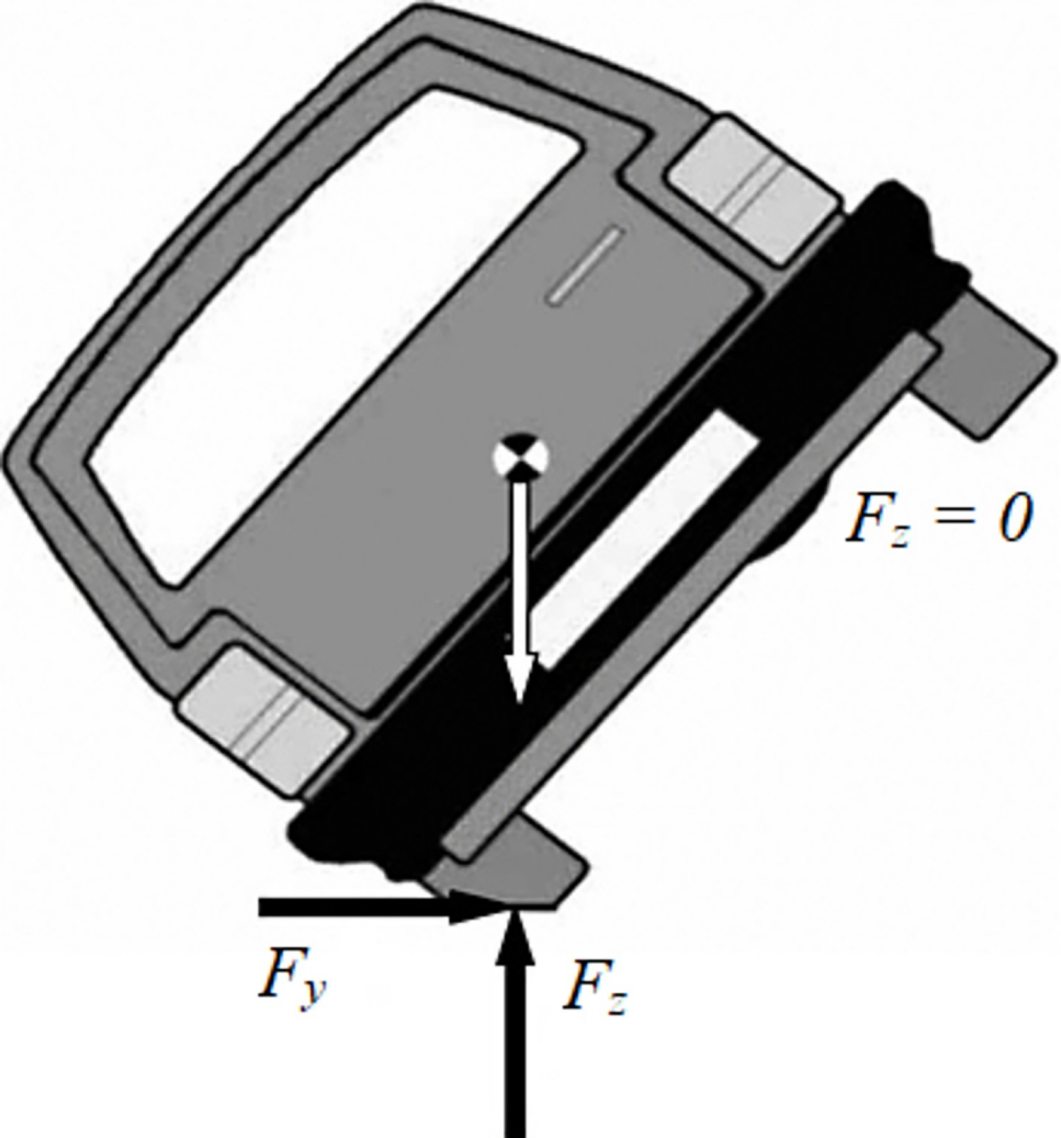
A vehicle rollover stability assessment scheme.

Rollovers are among the most dangerous accidents that frequently happen while an automobile is moving on the road. For developed and developing countries, the number of rollover accidents is increasing day by day. Rollover occurs when the wheels on the same side are completely separated from the road, i.e., the vertical force at the wheels will approach zero (Fig 1). Once this occurs, the vehicle body’s roll angle will be maximized before the automobile enters a dangerous state [1]. However, this is true if and only if the vehicle body is tilted under the influence of centrifugal force during steering, and it still tends to increase the roll angle. In simpler terms, if the roll angle increases to a critical threshold, the vertical force at the wheel will decrease to zero. A rollover may occur if the vehicle roll angle continues to increase (because it has not reached its maximum value). In some other cases, such as suspension system oscillations, the vertical force at the wheel can be reduced to zero, making the wheel able to separate from the road but not roll over. In general, once the vertical force drops sharply, instability will occur. Many causes cause rollovers, which should be clearly identified to limit this. According to Han and Rho, rollover mechanisms can be divided into 2 types: tripped and untripped [2]. However, the tripped condition occurs more rarely than others. According to Zhao et al., vehicle size and load parameters can also affect rollover [3]. If the vehicle’s height is too high or the track width is quite small, the car may more easily roll over. Besides, external factors such as road surface conditions [4, 5], weather conditions [6], etc., also greatly influence this problem. In particular, the user’s operation is the main cause of the car’s rollover. For example, if a driver is operating a car at too high a speed and suddenly steered, the vehicle may roll over immediately. In general, the consequences of these accidents are very serious.

In some studies, several indicators are commonly used to assess rollovers, such as RI (Roll Index) or LTR (Load Transfer Ratio). In [7], Li et al. introduced the LTR index, which is used for dynamic modeling with eight degrees of freedom (DOFs). This indicator is determined based on the difference between the dynamic forces of the right and left wheels. The wheel’s vertical force change depends on the vehicle’s lateral acceleration, which occurs when the car is steering. Therefore, this index can be rewritten in a different form as Eq (1). The symbols used in Eq (1) are explained well in [7]. However, if only the parameters on the right side of Eq (1), such as *h*, *t*_*w*_, *a*_*y*_, *g*, and *ϕ*, are used, the calculation will be inaccurate because the mass and stiffness of the suspension system are ignored. The LTR index is also used for the dynamic model with three DOFs, which is shown in the paper by Chao et al. According to Chao et al., the values of LTR ranged from -1 to +1, and they divided these values into five respective steps to assess the danger of rollover [8]. If the value of this indicator fluctuates around the zero point, the car will be more stable. On the other hand, if this value changes too much, it could lead to instability and even a rollover if |RI| = 1 [9]. The 3 DOFs model described in [8] is quite simple. The authors have ignored the influence of pitch angle and the deformation of nonlinear tires.

$$LTR=\frac{F_{zr}-F_{zl}}{F_{zr}+F_{zl}}=\frac{2h}{t_{w}}(\frac{a_{y}}{g}+sin\phi )$$ (1)

Rollover can be warned using several methods to predict it. Zhu et al. introduced a new predictive model called the SVM (Support Vector Machine) Empirical Model in [10], and the accuracy of this model ranges from 70 ÷ 100%. This is a new approach, and it is quite complicated. According to the diagram of the algorithm (Fig 3 in [10]), this model has no feedback from the output. Vehicle rollover can also be predicted utilizing the neural network algorithm introduced by Sanchez et al. in [11]. This is done based on the optimization of the Lyapunov function. The dynamic model of the car is not clearly described in [11]. Sellami et al. investigated the characteristics of some vehicle rollover prediction models in [12]. According to them, this phenomenon is evaluated based on the probability of a car rollover, calculated according to the LTR index. This index is once again used to predict vehicle rollover through the study of Wang et al. [13]. However, only a simple dynamic model (a combination of a half model and a linear motion model) is used to describe the oscillation characteristics of the car. This model ignores many other influences that can alter values related to rollover oscillation. Besides, many methods are used to indicate the rollover phenomenon, which has also been shown in several papers [14–16]. As a result, this prediction can generate an alert signal sent to the driver’s smartphone [17]. Therefore, the user can actively control the vehicle to limit the accident risk of rolling over.

Many modern mechatronic systems are used in cars to prevent rollover more effectively. For instance, using an active stabilizer bar to replace conventional stabilizers is also highly effective in anti-rolling [18, 19]. Additionally, using a gyro stabilizer to limit rollover is an exciting solution presented by Zhang et al. in [20]. Besides, the combined use of active steering [21, 22], active braking [23, 24], active suspension [25–27], and several other active control systems also helps to improve this problem [28–30]. Tires play an important role in ensuring the car’s stability when steering. Determining the forces at the tire is essential. Many methods are used to accomplish this, such as estimating the tire slip angle [31] or forces [32, 33].

Most studies on car rollover oscillations use only a simple dynamic model instead of a full model. Besides, nonlinear tire deformation is often ignored, although this is a significant issue directly related to rollover oscillations. In addition, the dependence between factors such as velocity, RA to CG distance, roll angle, and vertical force can only be described as 2D or 3D graphs without the simultaneous description of all four above values. Therefore, it is necessary to determine the correlation between these four factors in the form of a 4D graph. In this paper, the author proposes a new method to assess the dependence of vehicle rollover on other parameters. The paper’s results are presented in the form of 4D graphs instead of traditional graphs, which is considered a novelty and unique point of this paper. The model of a complex dynamic that combines multiple nonlinear components used to simulate vehicle oscillations under many driving conditions. The results obtained from the simulation process help verify the dependence between the dynamic force and the roll angle on the speed and size of the vehicle. These results are displayed on the same graph, which helps the reader better understand their relationship. These results are considered the basis for the future development of complex rollover prediction models, so they are considered the paper’s main contribution. The main content of this paper is divided into four parts, including an introduction section, a method section, a simulation section, and a conclusion section. Specific content will be presented in the following parts.

## 2. Method

Analytical and simulation methods are used in this paper. A model of dynamic must be set up in order to simulate vehicle oscillations during steering. The model of A complex dynamic is used in this study. This is a combination of three nonlinear dynamic models, including the tire model, the motion model, and the body oscillation model.

**Fig 2:**
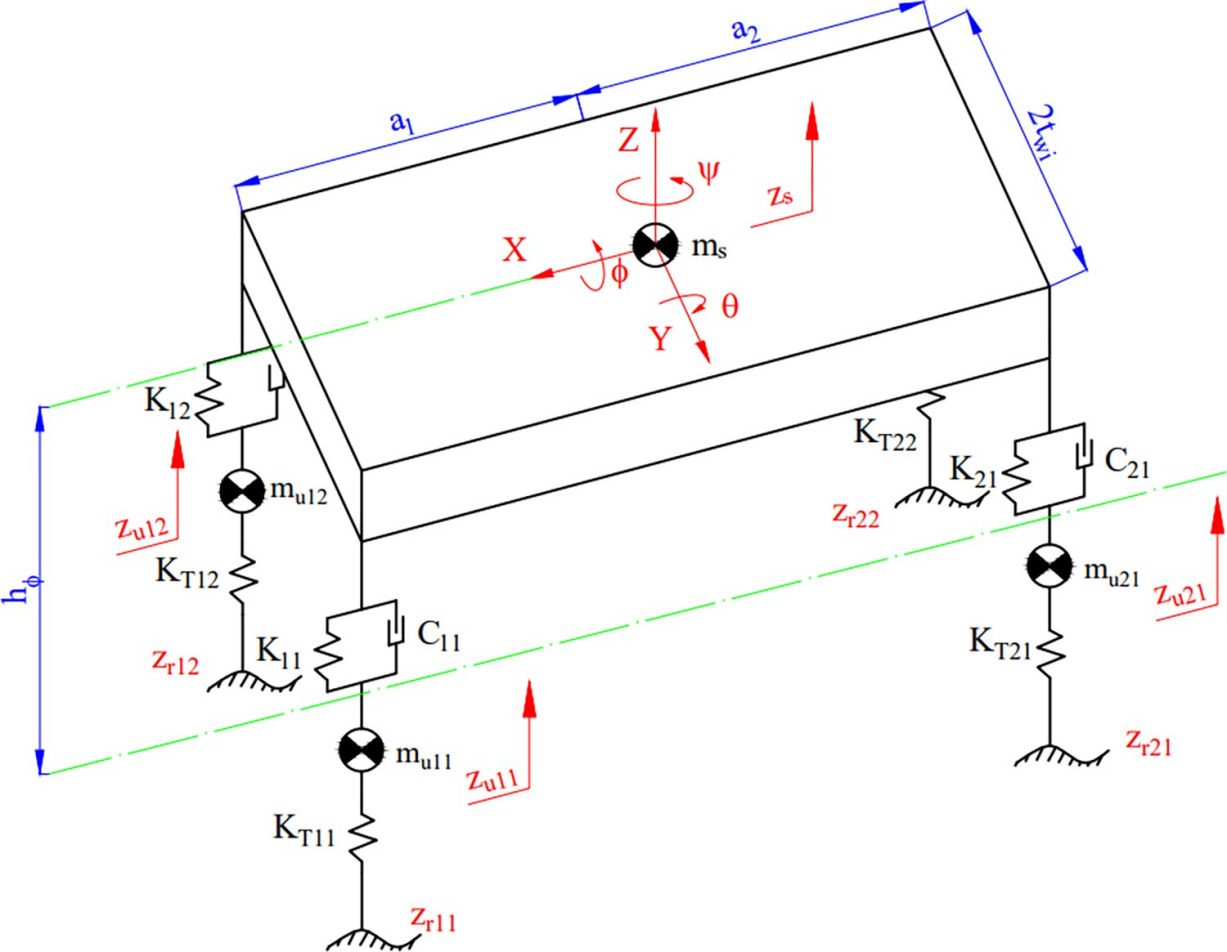
The vehicle oscillation model.

Consider the oscillation model of the vehicle body with five masses, including four unsprung masses and one sprung mass (Fig 2). Eqs from (2) to (12) describe the oscillations of these masses according to D’Alembert’s principle.

The force balance equation for the vehicle body (sprung mass):

$$F_{ims}=\sum_{i,j=1}^{2}\left(F_{Kij}+F_{Cij}\right)$$ (2)

The moment equilibrium equation for the vehicle body in the roll direction:

$$M_{\phi }=\sum_{i,j=1}^{2}\left[{\left(-1\right)}^{j-1}(F_{Kij}+F_{Cij})t_{wi}\right]+\{gsin\phi +[{\dot{v}}_{y}+(\dot{\beta }+\dot{\psi })v_{x}]cos\phi \}m_{s}h_{\phi }$$ (3)

The moment equilibrium equation for the vehicle body in the pitch direction:

$$M_{\theta }=\sum_{i,j=1}^{2}{\left(-1\right)}^{i-1}(F_{Kij}+F_{Cij})a_{i}$$ (4)

The force balance equations for the unsprung mass:

$$F_{imuij}=F_{KTij}-F_{Kij}-F_{Cij}\;i,j=\bar{1,2}$$ (5)

Where:

Force of inertia of the sprung mass, *F*_*ims*_:

$$F_{ims}=m_{s}{\ddot{z}}_{s}\;i,j=\bar{1,2}$$ (6)

Force of inertia of the unsprung mass, *F*_*imuij*_:

$$F_{imuij}=m_{uij}{\ddot{z}}_{uij}\;i,j=\bar{1,2}$$ (7)

The elastic force of spring, *F*_*Kij*_:

$$F_{Kij}=K_{ij}[z_{s}-z_{uij}+{\left(-1\right)}^{j+1}t_{wi}\phi +{\left(-1\right)}^{i+1}a_{i}\theta ]\;i,j=\bar{1,2}$$ (8)

The damping force of damper, *F*_*Cij*_:

$$F_{Cij}=C_{ij}[{\dot{z}}_{s}-{\dot{z}}_{uij}+{\left(-1\right)}^{j+1}t_{wi}\dot{\phi }+{\left(-1\right)}^{i+1}a_{i}\dot{\theta }]\;i,j=\bar{1,2}$$ (9)

The elastic force of tire, *F*_*KTij*_:

$$F_{KTij}=K_{Tij}(z_{rij}-z_{uij})\;i,j=\bar{1,2}$$ (10)

Moment of inertia of the vehicle body in the roll direction, *Mϕ*:

$$M_{\phi }=(J_{\phi }+m_{s}h_{\phi }^{2})\ddot{\phi }$$ (11)

Moment of inertia of the vehicle body in the pitch direction, *M*_*θ*_:

$$M_{\theta }=(J_{\theta }+m_{s}h_{\theta }^{2})\ddot{\theta }$$ (12)

**Fig 3:**
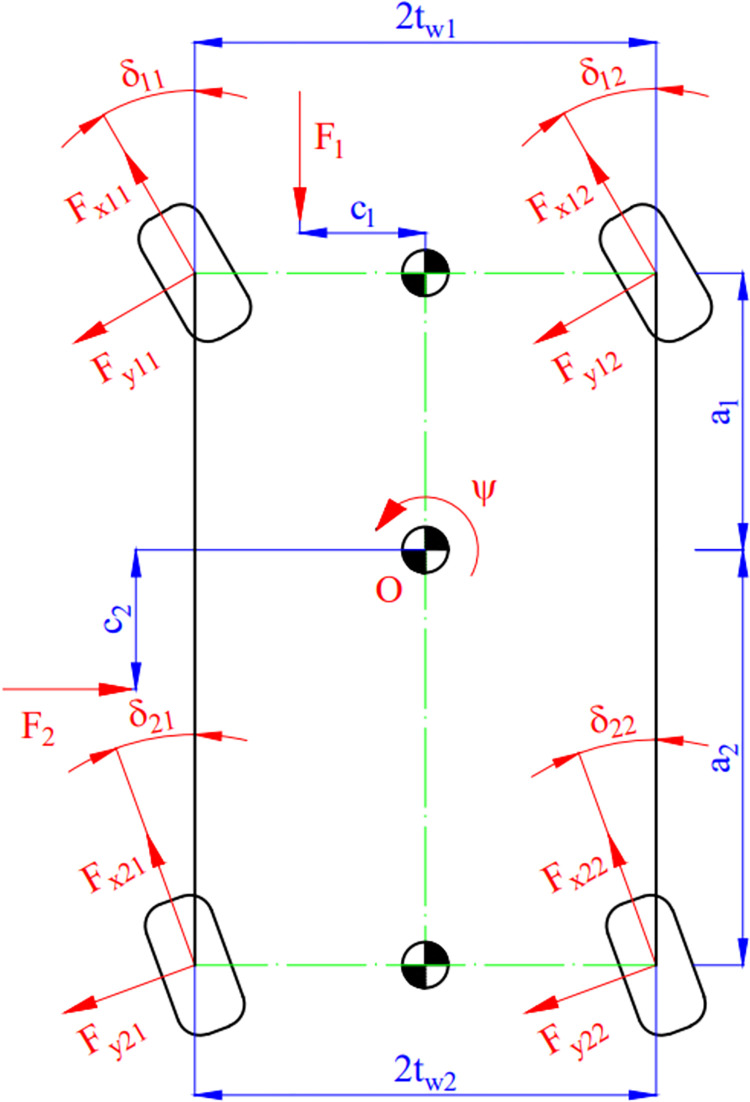
The vehicle motion model.

The components of lateral velocity *v*_*y*_, longitudinal velocity *v*_*x*_, and yaw angle *ψ* in the Eq (3) can be determined by the motion model with three DOFs (Fig 3). This nonlinear model is described by differential Eqs (13) to (15).

The force balance equation in the longitudinal direction:

$$F_{ix}=\sum_{i,j=1}^{2}\left(F_{xij}cos\delta_{ij}-F_{yij}sin\delta_{ij}\right)-F_{1}+F_{cex}$$ (13)

The force balance equation in the lateral direction:

$$F_{iy}=\sum_{i,j=1}^{2}\left(F_{xij}sin\delta_{ij}+F_{yij}cos\delta_{ij}\right)-F_{2}-F_{cey}$$ (14)

The moment balance equation of the vehicle:

$$M_{\psi }=\sum_{i,j=1}^{2}\left[{\left(-1\right)}^{j}(F_{xij}cos\delta_{ij}-F_{yij}sin\delta_{ij})t_{wi}+{\left(-1\right)}^{i+1}(F_{xij}sin\delta_{ij}+F_{yij}cos\delta_{ij})a_{i}+F_{i}c_{i}-M_{zij}\right]$$ (15)

Where:

Force of inertia in the longitudinal direction:

$$F_{ix}=(m_{s}+\sum_{i,j=1}^{2}m_{uij}){\dot{v}}_{x}$$ (16)

Force of inertia in the lateral direction:

$$F_{iy}=(m_{s}+\sum_{i,j=1}^{2}m_{uij}){\dot{v}}_{y}$$ (17)

Centrifugal force in the longitudinal direction:

$$F_{cex}=(m_{s}+\sum_{i,j=1}^{2}m_{uij})\left(\dot{\beta }+\dot{\psi }\right)v_{y}$$ (18)

Centrifugal force in the lateral direction:

$$F_{cey}=(m_{s}+\sum_{i,j=1}^{2}m_{uij})\left(\dot{\beta }+\dot{\psi }\right)v_{x}$$ (19)

Moment of inertia of the vehicle:

$$M_{\psi }=J_{\psi }\ddot{\psi }$$ (20)

Heading angle of the vehicle:

$$\beta =arctan\frac{v_{y}}{v_{x}}$$ (21)

In Eqs (14) to (16), unknowns such as the lateral force at the wheels, *F*_*yij*_, the longitudinal force at the wheels, *F*_*xij*_, and the moment at the wheels, *M*_*zij*_, have not been determined. These parameters can be calculated by the tire model. Many types of tire models are commonly used in studies of automobile motion, such as linear tire models, Burckhardt tire models, Ammon tire models, etc. In particular, the Pacejka tire model that is utilized in this paper has many outstanding advantages. However, this model is quite complicated, it is shown by Eqs from (22) to (46).

Longitudinal force *F*_*x*_:

$$F_{x}=D_{x}sin[C_{x}artan(B_{x}\kappa_{x})]$$ (22)

Where:

$$\kappa_{x}=\left(1-E_{x}\right)\lambda +\frac{E_{x}}{B_{x}}arctan(B_{x}\lambda )$$ (23)

$$C_{x}=1.65$$ (24)

$$D_{x}=a_{1}F_{z}^{2}+a_{2}F_{z}$$ (25)

$$BCD_{x}=\frac{a_{3}F_{z}^{2}+a_{4}F_{z}}{e^{a_{5}F_{z}}}$$ (26)

$$B_{x}=\frac{BCD_{x}}{C_{x}D_{x}}$$ (27)

$$E_{x}=a_{6}F_{z}^{2}+a_{7}F_{z}+a_{8}$$ (28)

Lateral force *F*_*y*_:

$$F_{y}=D_{y}sin[C_{y}artan(B_{y}\kappa_{y})]+S_{vy}$$ (29)

Where:

$$\kappa_{y}=\left(1-E_{y}\right)\left(\alpha +S_{hy}\right)+\frac{E_{y}}{B_{y}}arctan\left[B_{y}(\alpha +S_{hy}\right)]$$ (30)

$$C_{y}=1.30$$ (31)

$$D_{y}=a_{1}F_{z}^{2}+a_{2}F_{z}$$ (32)

$$BCD_{y}=a_{3}sin[a_{4}artan(a_{5}F_{z})]$$ (33)

$$B_{y}=\frac{BCD_{y}}{C_{y}D_{y}}$$ (34)

$$E_{y}=a_{6}F_{z}^{2}+a_{7}F_{z}+a_{8}$$ (35)

$$S_{hy}=a_{9}\gamma$$ (36)

$$S_{vy}=(a_{10}F_{z}^{2}+a_{11}F_{z})\gamma$$ (37)

Aligning moment *M*_*z*_:

$$M_{z}=D_{z}sin[C_{z}artan(B_{z}\kappa_{z})]+S_{vz}$$ (38)

Where:

$$\kappa_{z}=\left(1-E_{z}\right)\left(\alpha +S_{hz}\right)+\frac{E_{z}}{B_{z}}arctan\left[B_{z}(\alpha +S_{hz})\right]$$ (39)

$$C_{z}=2.40$$ (40)

$$D_{z}=a_{1}F_{z}^{2}+a_{2}F_{z}$$ (41)

$$BCD_{z}=\frac{a_{3}F_{z}^{2}+a_{4}F_{z}}{e^{a_{5}F_{z}}}$$ (42)

$$B_{z}=\frac{BCD_{z}}{C_{z}D_{z}}$$ (43)

$$E_{z}=a_{6}F_{z}^{2}+a_{7}F_{z}+a_{8}$$ (44)

$$S_{hz}=a_{9}\gamma$$ (45)

$$S_{vz}=(a_{10}F_{z}^{2}+a_{11}F_{z})\gamma$$ (46)

**Fig 4:**
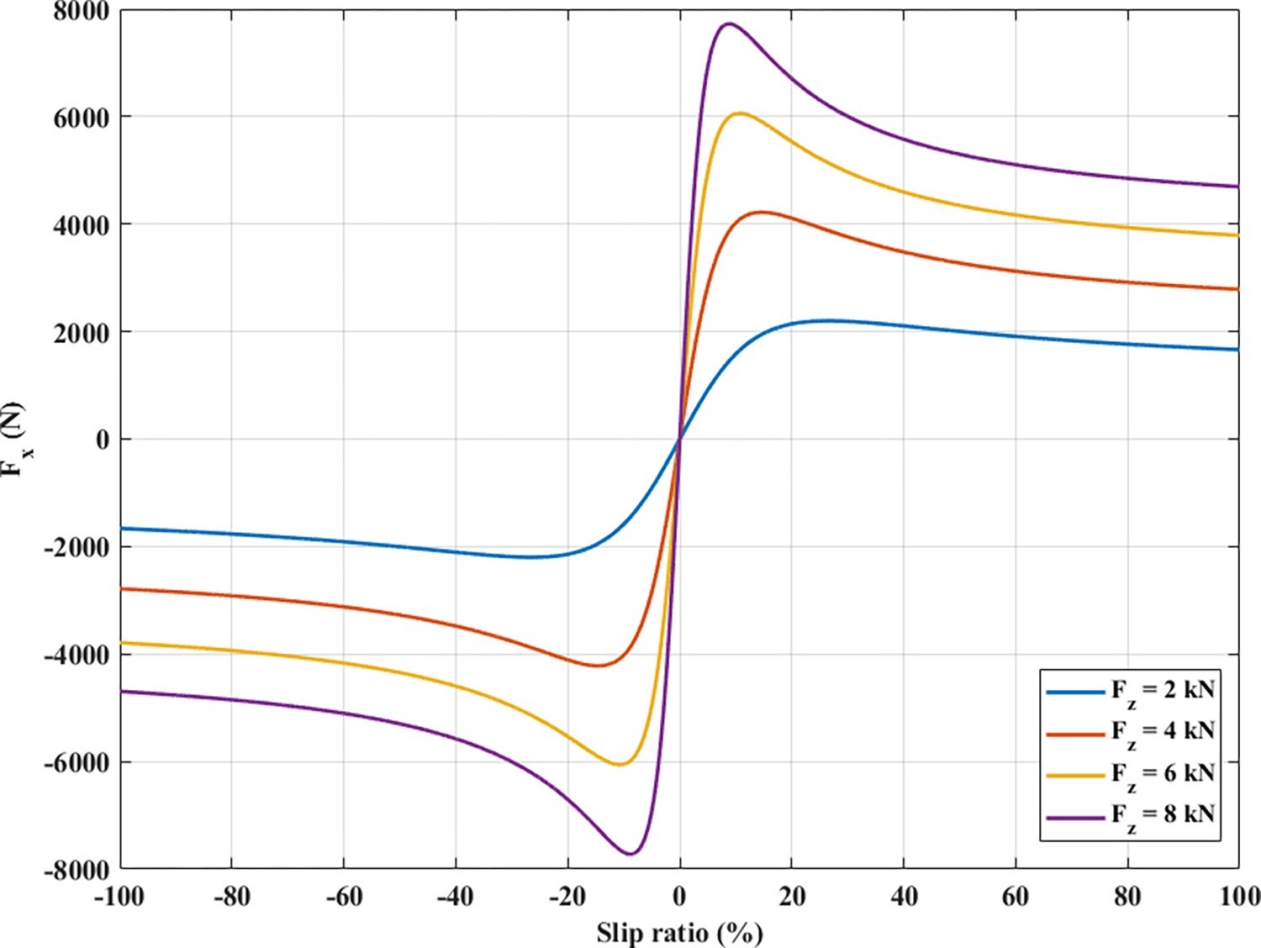
The longitudinal force.

**Fig 5:**
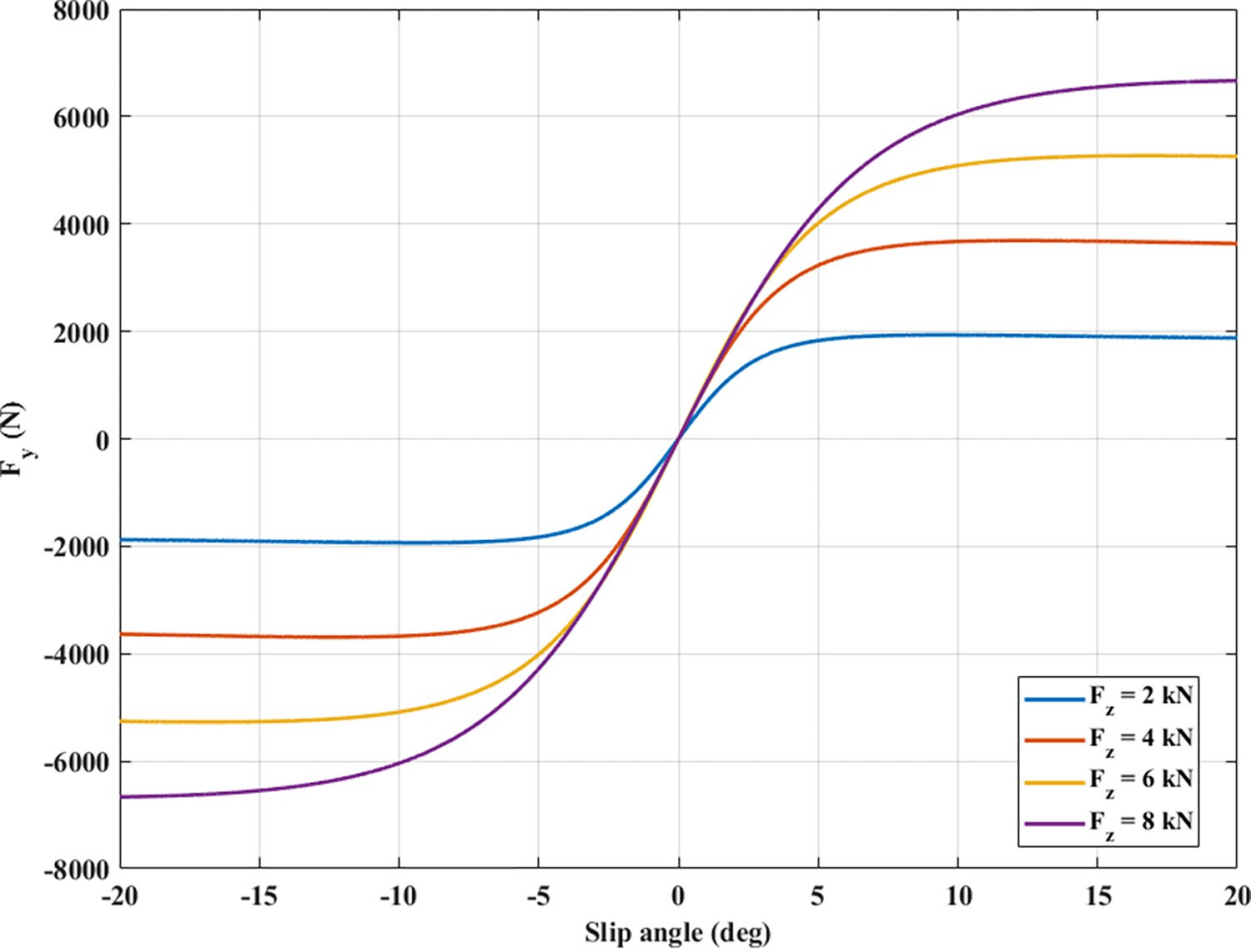
The lateral force.

The Pacejka tire model needs to use many experimental parameters. A tire’s longitudinal and lateral forces are functions of the wheel’s slip ratio and angle. This relationship can be observed in Figs 4 and 5.

## 3. Simulation

**Fig 6:**
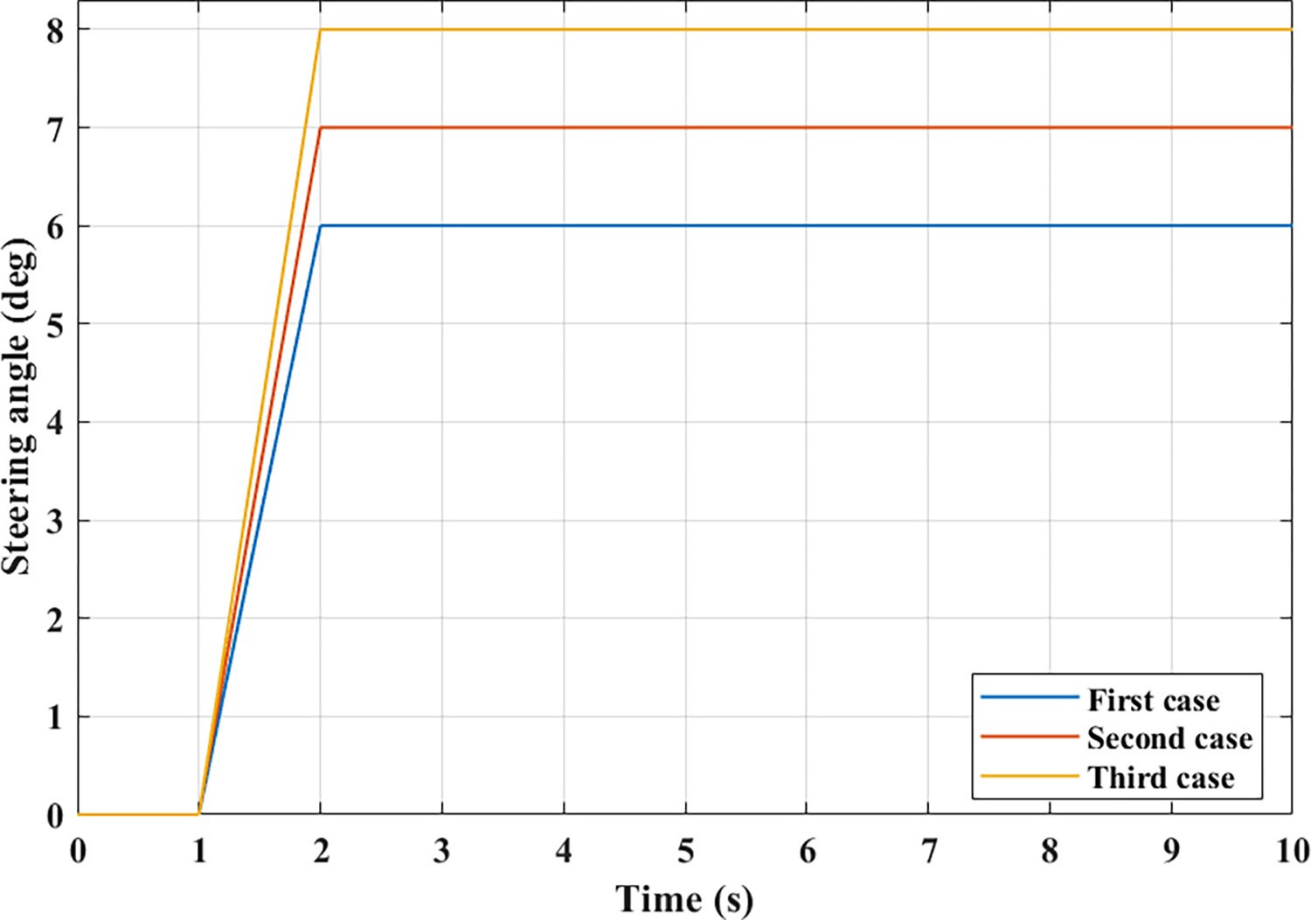
Steering angle.

The simulation process is done after establishing the model of complex dynamics. This process occurs in the real-time domain in the Simulink^®^ environment. The input to the simulation problem includes the change of steering angle, motion velocity, and distance from CG to RA. The output of the simulation problem consists of the change of the roll angle and the dynamic force at the wheel. Three cases are used to simulate the three steering angles (Fig 6). This is the J-turn type of steering, which is commonly used in practice when the car enters a roundabout. From the first case to the third case, both the steering angle and the angular acceleration get bigger.

The vehicle specifications utilized in the simulation are shown in Table 1.

**Table 1:** The automobile specifications.

No	Description	Symbol	Unit	Value
1	Sprung mass	m_s_	kg	1892
2	Unsprung mass	m_u_	kg	40
3	Half the front track width	t_w1_	mm	800
4	Half the rear track width	t_w2_	mm	810
5	Spring stiffness of the front axle	K_11_/K_12_	Nm^-1^	46200
6	Spring stiffness of the rear axle	K_21_/K_22_	Nm^-1^	45600
7	Spring tire of the front axle	K_T11_/K_T12_	Nm^-1^	178500
8	Spring tire of the rear axle	K_T21_/K_T22_	Nm^-1^	178500
9	Damper coefficient of the front axle	C_11_/C_12_	Nsm^-1^	3360
10	Damper coefficient of the rear axle	C_21_/C_22_	Nsm^-1^	3240

**Fig 7:**
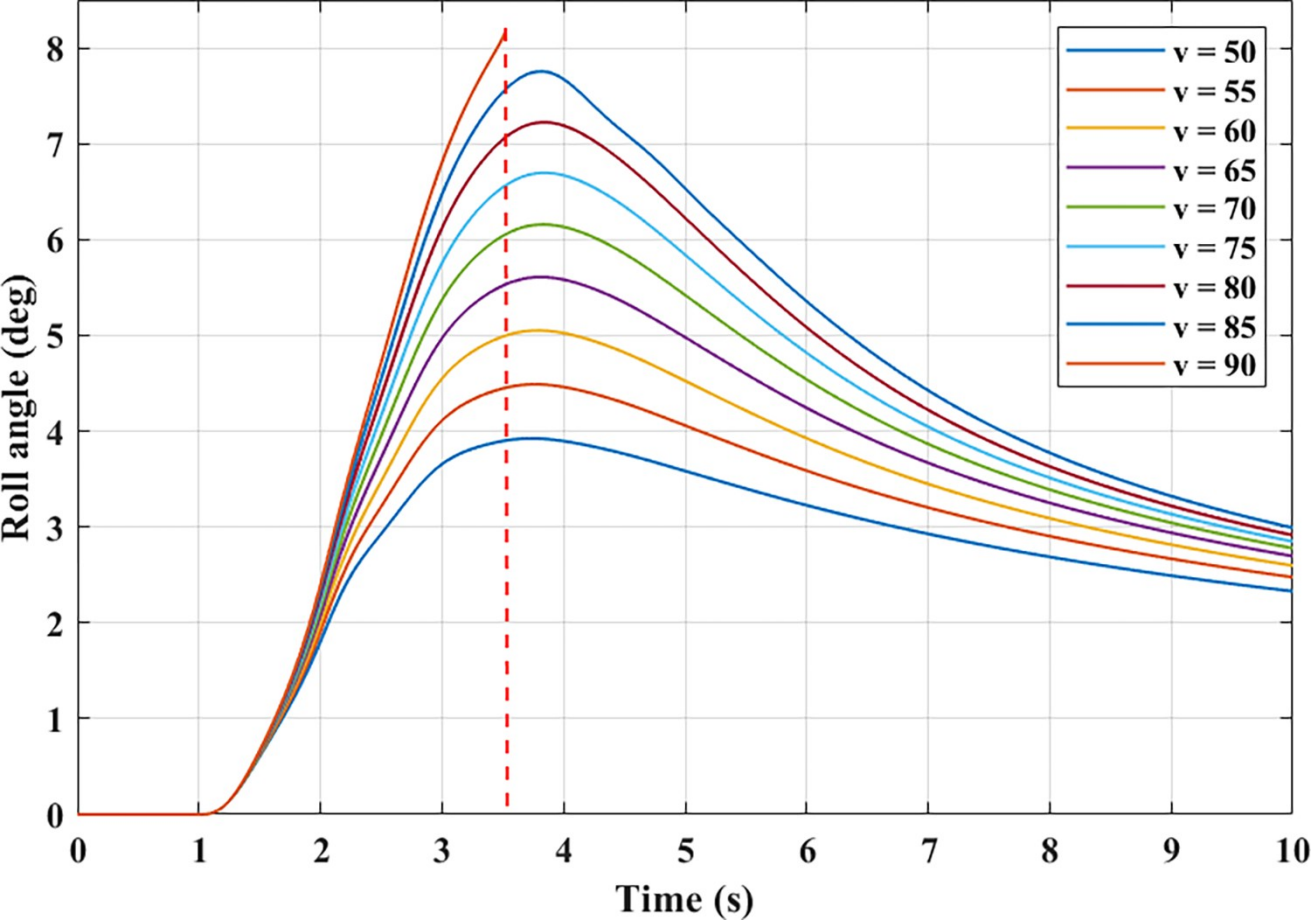
The relationship between velocity and roll angle.

The roll angle also changes as the vehicle’s velocity changes with time. This relationship is shown in Fig 7. Since all three cases (Fig 6) give similar graphs (different only in the amplitude), the first case is chosen to illustrate this dependency. Based on the results obtained from the simulation, roll angle increases as the velocity increases. This change is nonlinear and can only be determined through the model of complex dynamics established above. In the first phase of the steering process, the roll angle increases to its maximum value, reaching 3.92°, 4.48°, 5.05°, 5.61°, and 6.16°, respectively, at speeds from 50 (km/h) to 70 (km/h). This value can further increase to 6.69°, 7.22°, and 7.75° if the vehicle velocity reaches 75 (km/h), 80 (km/h), and 85 (km/h). Once the vehicle travels at a dangerous speed, *v* = 90 (km/h), a rollover can occur (the vertical force at the wheel was zero). The limited roll angle of the car at this time is only 8.11°, and it has not even reached the peak value corresponding to this speed. The steering angle is unchanged in the second phase of the steering process. Under the influence of the nonlinear tire model, the vehicle’s trajectory will change even though the speed and steering angle remains the same. In this situation, the vehicle’s trajectory will increase (understeering), which will decrease the body’s roll angle. The values of the roll angle corresponding to the velocity values tend to decrease. If the velocity is greater, the attenuation will also be greater.

**Fig 8:**
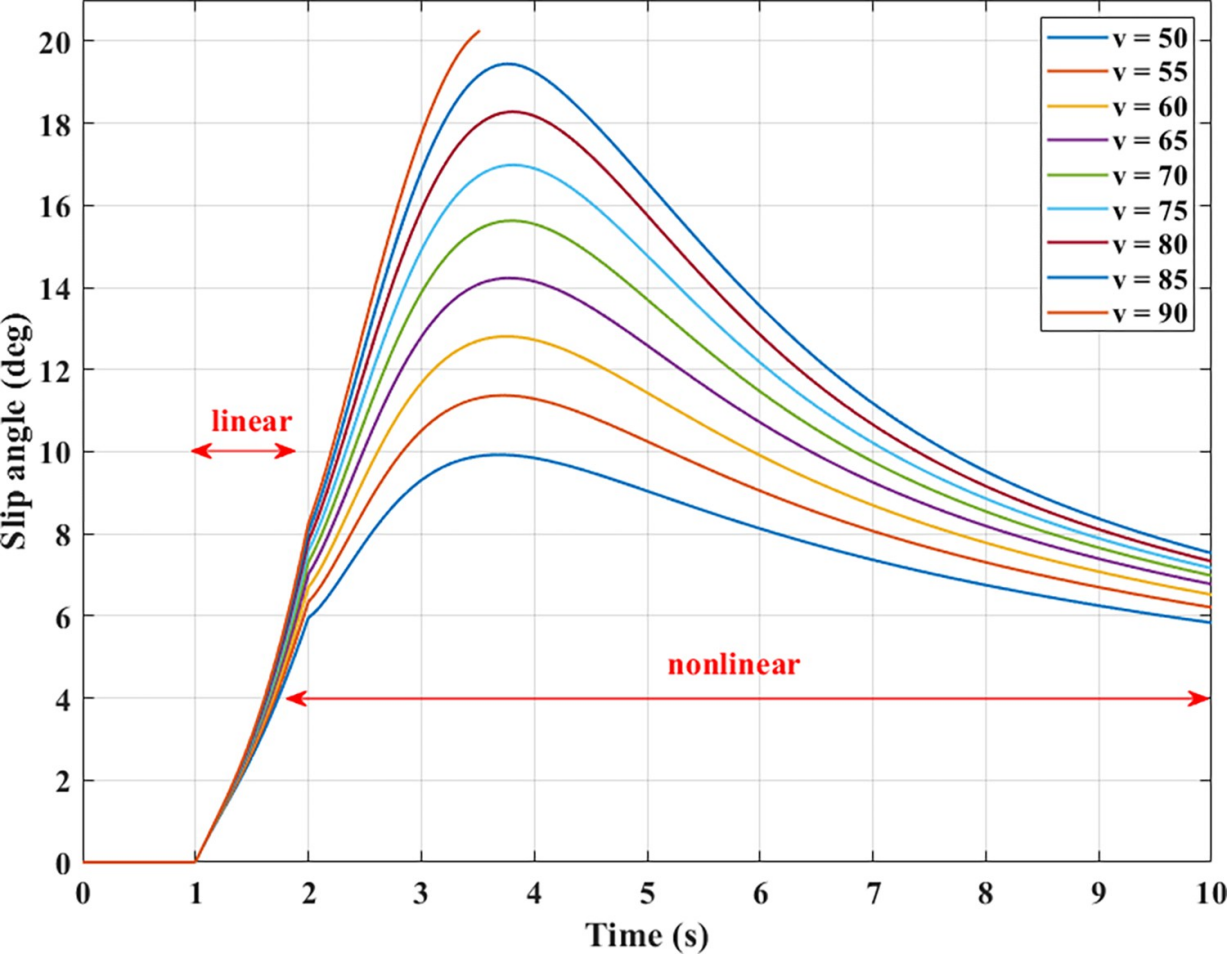
Slip angle.

Tire deformation is described through the change in slip angle. According to the results illustrated in Fig 8, increasing the slip angle is considered linear when its values are small. If this value increases beyond the limited threshold, its change is nonlinear. This causes the vehicle’s roll angle to decrease in the second phase, although the steering angle remains the same. The value of the slip angle is proportional to the velocity according to a nonlinear function that cannot be determined precisely by simple formulas. Once the velocity is increased, the change in slip angle is more significant.

**Fig 9:**
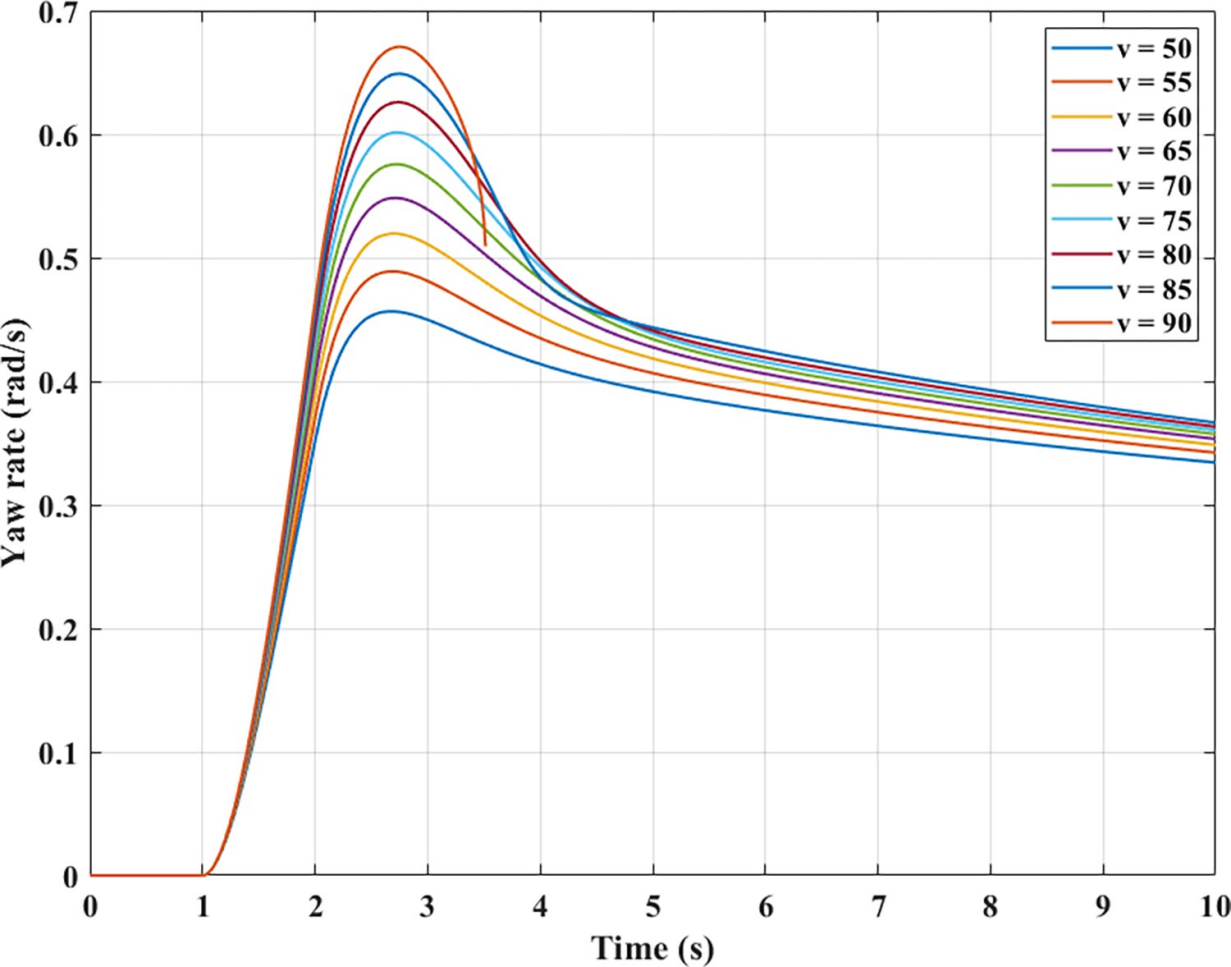
Yaw rate.

In vehicle dynamics studies, the yaw rate value is also often considered. According to the results shown in Fig 9, the yaw rate value increases rapidly when steering. After that, it tends to decrease over time (similar to the roll and slip angles). If the speed increases, the yaw rate will also be more significant. If this value changes too quickly or is too large, it can cause yaw instability, i.e., change the direction of motion of the car.

**Fig 10:**
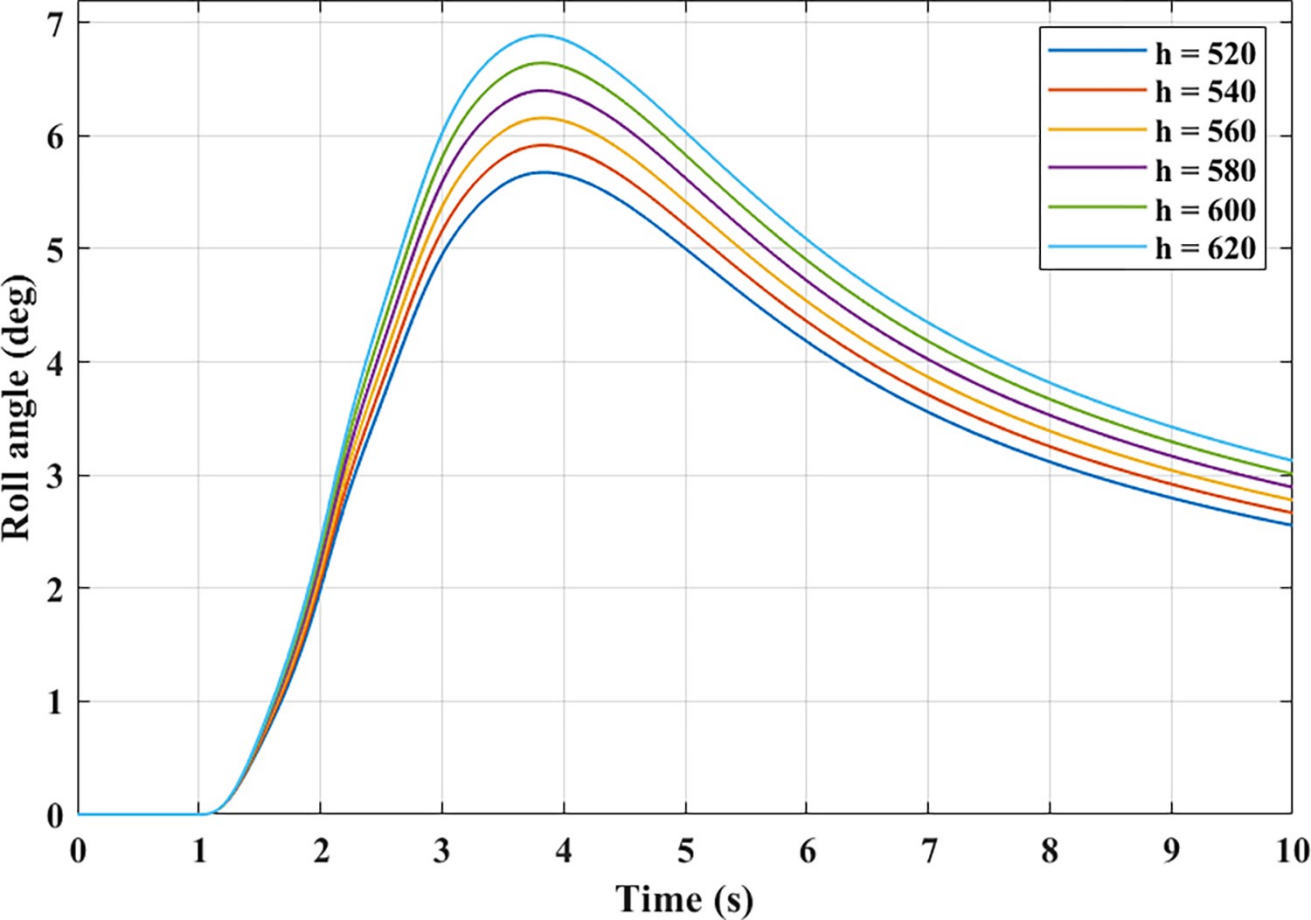
The relationship between height and roll angle.

Even though the vehicle’s speed remains the same, the roll angle still changes if the distance from CG to RA changes. The dependence between these two objects is illustrated in Fig 10. Based on this finding, the larger the distance *h*, the larger the value of the roll angle will be. The peak value of the roll angle is reached at the end of the first phase, after which it decreases with time. The difference between the two results is quite significant, up to 1.21°, corresponding to *h* = 520 (mm) and *h* = 620 (mm). The change in slip angle and yaw rate in the case of a change in height is similar to that in the case of a change in velocity. However, their variation is more minor.

**Fig 11:**
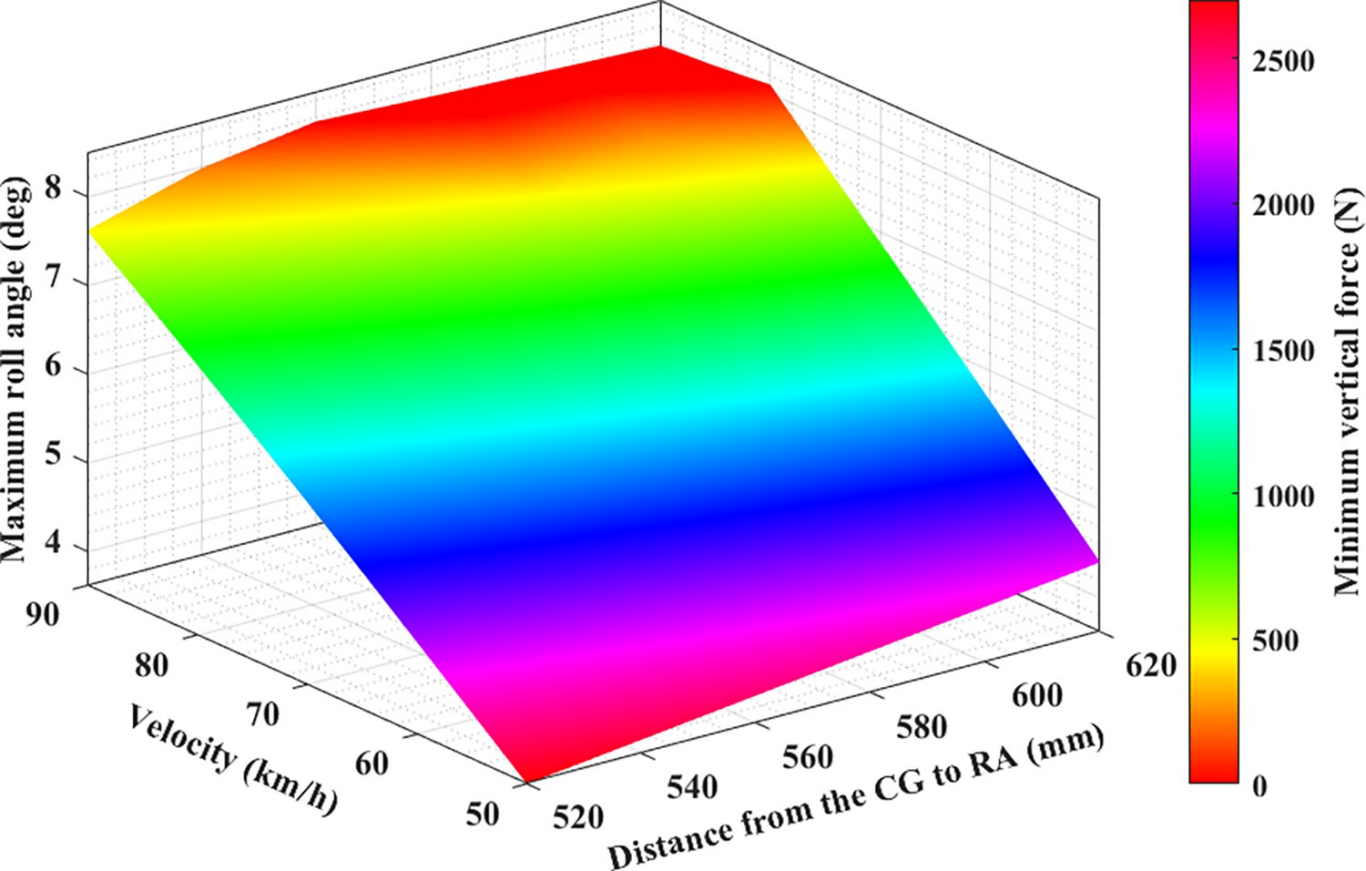
Limitation of rollover phenomenon (the first case).

**Fig 12:**
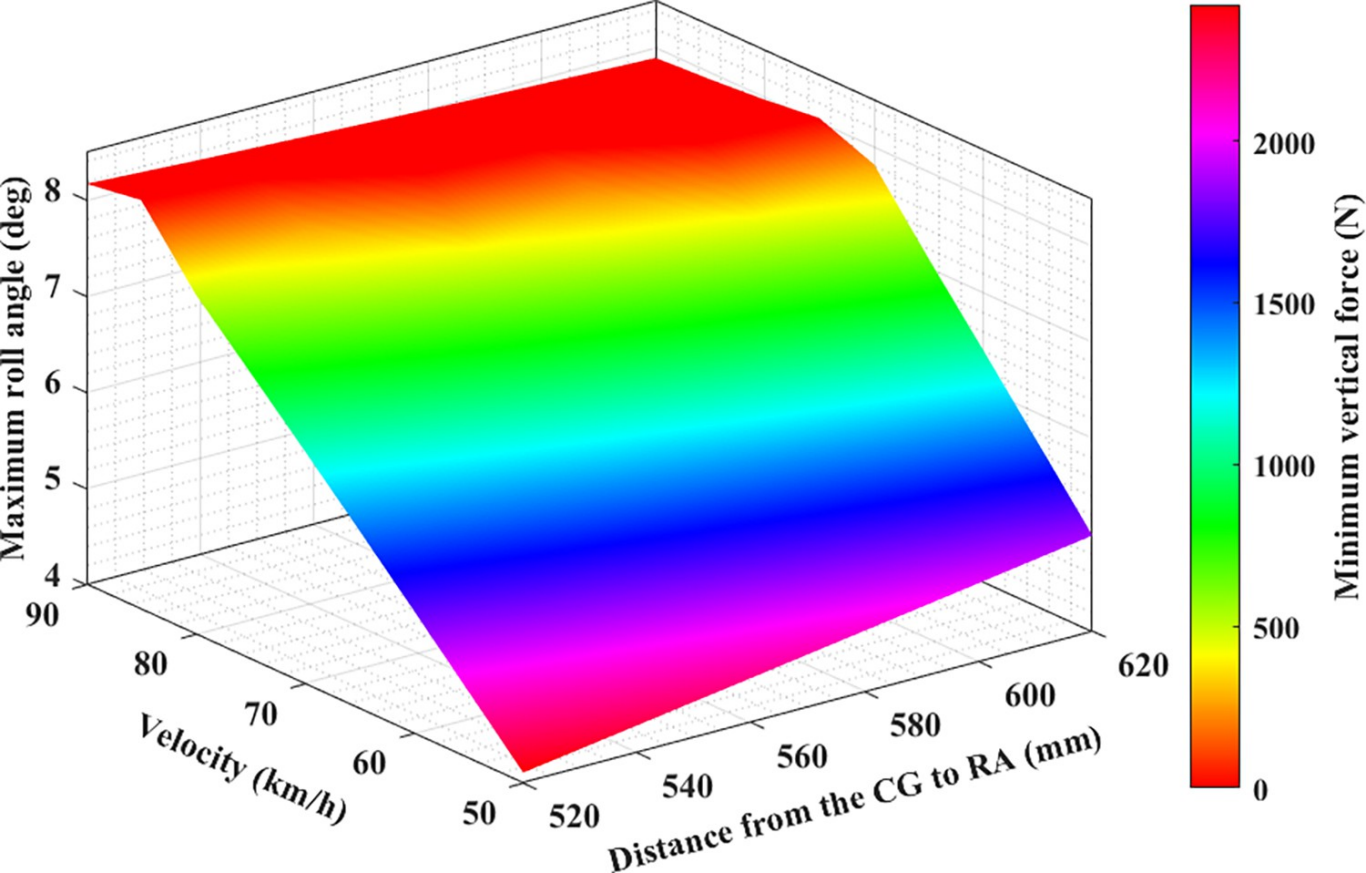
Limitation of rollover phenomenon (the second case).

**Fig 13:**
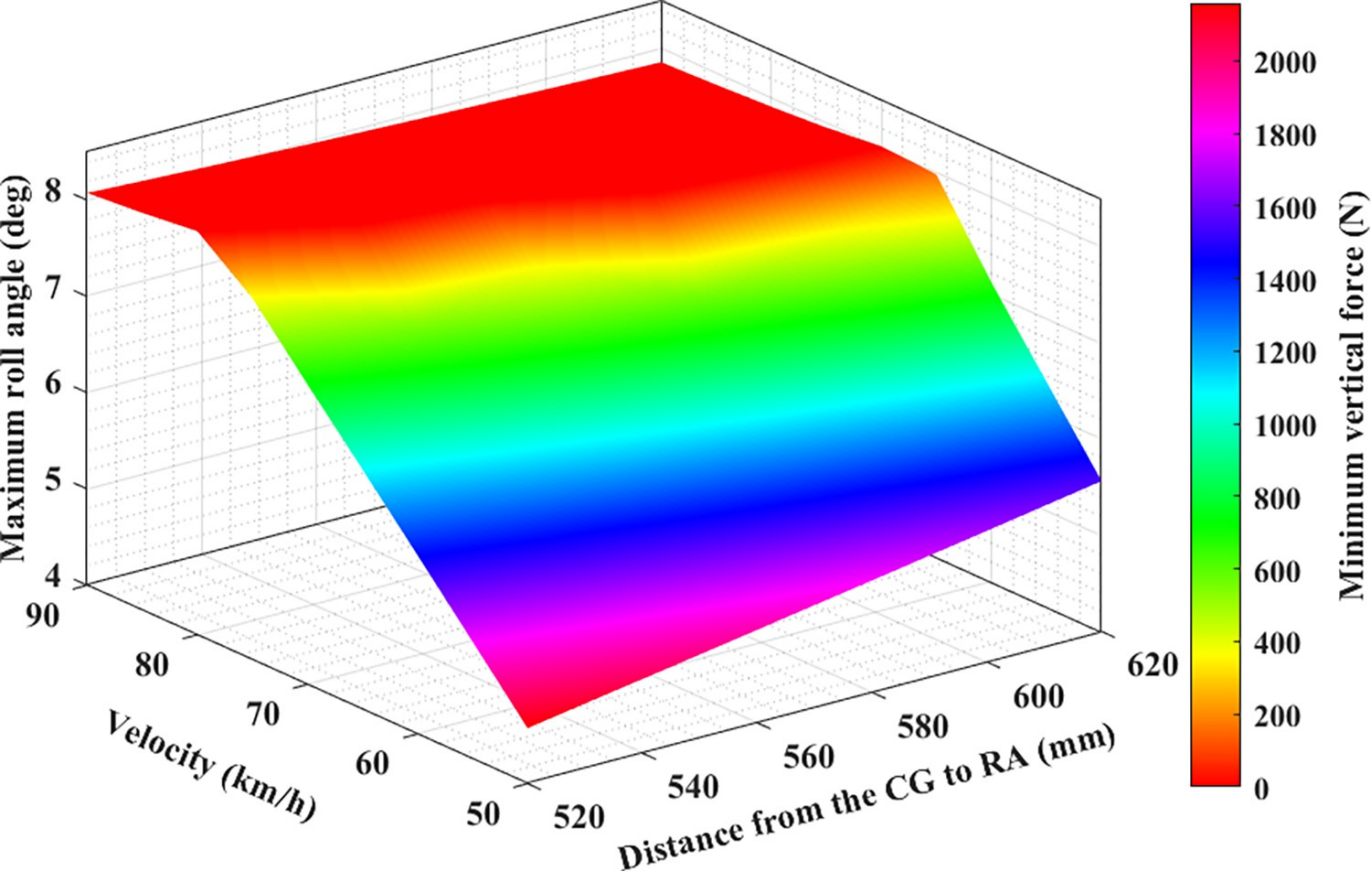
Limitation of rollover phenomenon (the third case).

In this paper, a new result was found: the dependence between the minimum dynamic force and the maximum roll angle of the wheel on the speed and distance from CG to RA. This result is presented in four-dimensional graphs in Figs 11–13. Fig 11 depicts the results obtained in the first case. According to this result, if the distance from CG to RA does not change, the maximum roll angle will increase if the speed increases. This increase is considered to be almost linear. Besides, if the speed is not changed, the maximum roll angle will also increase as the distance increases, which is also considered almost linear. However, if speed and distance increase, the maximum roll angle will rise more. This change is nonlinear, and it is difficult to determine precisely. This is only true when the vehicle moves steadily, and a rollover does not occur. If a rollover occurs, the limited roll angle will decrease as speed and distance increase. This can be understood simply by saying that if the vehicle is large and moving at high speed, it will be easier for the car to roll over. The change of the minimum force value at the wheel is opposite to the maximum roll angle. If the maximum roll angle is larger, the minimum force of the wheel will decrease. Once this value reduces to zero, the rollover will occur.

The dependence between the maximum roll angle, the minimum force at the wheel, and the speed and height in the second and third cases is illustrated in Figs 12 and 13, respectively. In these two cases, the values of the steering angle and the steering acceleration increase, so the maximum roll angle will be larger than in the first case with the same speed and height conditions (this is only true when rollover does not occur). Besides, the risk of rolling over in these two cases is higher than in the first. This implies that the limited roll angle can decline even if the vehicle speed is slightly above 75 (km/h) (Fig 11). The rollover warning limit can be observed more clearly in the red plot area, where it is shown that the dynamic force at the wheel is approaching zero. Compared to the conventional 2D or 3D graphs, the 4D graphs shown in this paper can provide better performance when assessing the rollover phenomenon.

Obviously, the greater the speed, height, and the steering angle, the greater the vehicle’s roll angle. Additionally, the value of the dynamic force at the wheel will decrease more, and the phenomenon of rolling over can occur suddenly. It is necessary to direct the vehicle at the reasonable speed and steering angle to limit this situation. Besides, the dimensions of the car also need to be optimally calculated.

## 4. Conclusion

There are many risks when an automobile moves on the road, in which the phenomenon of vehicle rollover is considered one of the most dangerous problems. This phenomenon is caused by many reasons, including the vehicle’s size parameters and the driver’s usage. In this study, the author introduced a new method to evaluate the factors affecting the rollover phenomenon comprehensively. The model of a complex dynamic that combines the Pacejka nonlinear tire model, the nonlinear motion dynamics model, and the spatial model was used to describe vehicle’s oscillations during the steering process. Instead of conventional 2D or 3D graphs, new 4D graphs were utilized to describe the dependence between the maximum roll angle and the minimum dynamic force at the wheel on the velocity and distance from the CG to RA. The simulation is performed with three specific cases in the Simulink^®^ environment.

The study’s results show that the roll angle’s value will increase as the speed goes up. Furthermore, the roll angle value increases as the distance h increases. These increases are relatively even if the interval is even. The larger the roll angle, the stronger the attenuation of the vertical force at the wheel. Once this value drops to zero, instability can occur. The results shown on the 4D graphs show that the vertical force at the wheel decreases very sharply once both the vehicle speed and height increase. However, if the vehicle rolls over (while traveling at high speeds and making sharp turns), the limited roll angle will decrease as the vehicle speed or size increases (because the vehicle rolls over before the roll angle reaches its maximum value). The relationship between the maximum roll angle, wheel dynamics, distance, and speed are clearly depicted on the 4D graphs that are obtained from the simulation. These graphs are seen as a new tool to help more intuitively assess problems related to automotive dynamics.

**Table:** Abbreviations

Symbol	Description	Unit	Symbol	Description	Unit
ϕ	Roll angle	rad	F_zij_	Vertical force	N
ψ	Yaw angle	rad	h_ϕ_	Distance from CG to RA	m
θ	Pitch angle	rad	h_θ_	Distance from CG to PA	m
β	Slip angle	rad	J_ϕ_	Roll inertia moment	kgm^2^
δ_ij_	Steering angle	rad	J_θ_	Pitch inertia moment	kgm^2^
a_i_	Distance from CG to axles	m	J_ψ_	Yaw inertia moment	kgm^2^
F_1_/F_2_	External force	N	Mϕ	Roll moment	Nm
F_cex_	Longitudinal centrifugal force	N	M_θ_	Pitch moment	Nm
F_cey_	Lateral centrifugal force	N	M_ψ_	Yaw moment	Nm
F_Cij_	Damping force	N	m_s_	Sprung mass	kg
F_ims_	Inertia force (sprung mass)	N	m_uij_	Unsprung mass	kg
F_imuij_	Inertia force (unsprung mass)	N	M_zij_	Aligning moment	Nm
F_ix_	Longitudinal inertia force	N	t_wi_	Half of track width	m
F_iy_	Lateral inertia force	N	v_x_	Longitudinal velocity	m/s
F_Kij_	Spring force	N	v_y_	Lateral velocity	m/s
F_KTij_	Tire force	N	z_rij_	Bump on the road	m
F_xij_	Longitudinal force	N	z_s_	Sprung mass displacement	m
F_yij_	Lateral force	N	z_uij_	Unsprung mass displacement	m
